# Evaluation of Myocardial Ischemia with iFR (Instantaneous Wave-Free Ratio in the Catheterization Laboratory: A Pilot Study

**DOI:** 10.36660/abc.20180298

**Published:** 2020-02

**Authors:** Heitor Cruz Alves Vieira, Maria Cristina Meira Ferreira, Leonardo Cruz Nunes, Carlos José Francisco Cardoso, Emilia Matos do Nascimento, Gláucia Maria Moraes de Oliveira

**Affiliations:** 1 Hospital Naval Marcilio Dias, Rio de janeiro, RJ - Brazil; 2 Universidade Federal do Rio de Janeiro, Rio de Janeiro, RJ - Brazil; 3 Hospital Federal dos Servidores do Estado, Rio de Janeiro, RJ - Brazil; 4 Fundação Centro Universitário Estadual da Zona Oeste - UEZO, Rio de Janeiro, RJ - Brazil

**Keywords:** Myocardial Ischemia, Fractional Flow Reserve Myocardial, Stents, Coronary Artery Disease, Risk factors, Percutaneous Coronary Intervention

## Abstract

**Background:**

The Instantaneous Wave-Free Ratio (iFR) is an invasive functional evaluation method that does not require vasoactive drugs to induce maximum hyperemia

**Objective:**

To evaluate the contribution of the iFR to the therapeutic decision-making of coronary lesions in the absence of non-invasive diagnostic methods for ischemia, or in case of discordance between these methods and coronary angiography.

**Method:**

We studied patients older than 18 years, of both sexes, consecutively referred for percutaneous treatment between May 2014 and March 2018. Coronary stenotic lesions were classified by visual estimation of the stenosis diameter into moderate (41-70% stenosis) or severe (71%-90%). An iFR ≤ 0.89 was considered positive for ischemia. Logistic regression was performed using the elastic net, with placement of stents as outcome variable, and age, sex, arterial hypertension, diabetes, dyslipidemia, smoking, family history, obesity and acute myocardial infarction (AMI) as independent variables. Classification trees, ROC curves, and Box Plot graphs were constructed using the R software. A p-value < 0.05 was considered statistically significant.

**Results:**

Fifty-two patients with 96 stenotic lesions (56 moderate, 40 severe) were evaluated. The iFR cut-off point of 0.87 showed a sensitivity of 0.57 and 1-specificity of 0.88, demonstrating high accuracy in reclassifying the lesions. Diabetes mellitus, dyslipidemia, and presence of moderate lesions with an iFR < 0.87 were predictors of stent implantation. Stents were used in 32% of lesions in patients with stable coronary artery disease and AMI with or without ST elevation (non-culprit lesions).

**Conclusion:**

The iFR has an additional value to the therapeutic decision making in moderate and severe coronary stenotic lesions, by contributing to the reclassification of lesions and decreasing the need for stenting.

## Introduction

In functional evaluation of coronary stenosis, the use of fractional flow reserve (FFR) to measure pressure instead of flow has been recommended by the American College of Cardiology-American Heart Association, the European Society of Cardiology, and the Brazilian Society of Hemodynamics and Interventional Cardiology guidelines^1-6^ in case of absence or inconclusive results from non-invasive methods to assess ischemia. FFR is an easy-to-perform technique and its efficacy has been demonstrated by several clinical trials, especially those on stable coronary artery disease patients. However, the FFR method is not widely used in clinical practice. One reason for that is that FFR is measured during maximal hyperemia, which is achieved by administration of vasodilator drugs (e.g. adenosine).^7^

The instantaneous wave-free ratio (iFR) is a recent, invasive method for functional diagnosis of coronary stenosis, introduced to solve some FFR-related issues, such as the need for intravenous drugs and new vascular access, with higher risk of complications.^8-10^ The comparison between these methods showed a strong correlation of iFR < 086 with positive FFR (≤ 0.80) for ischemia, and of iFR > 0.93 with negative FFR (FFR > 0.80) for ischemia, indicating the high accuracy of the method. Values of iFR located in the range of 0.86-0.93 (called the “grey-zone”) showed a weak correlation, and results were confirmed by FFR. This analysis using both iFR and FFR is known as a hybrid approach.^11,12^ The iFR was subsequently validated in randomized, controlled clinical trials which showed that the method was non-inferior to FFR, with cut-off points of 0.89 and 0.80 for iFR and FFR, respectively.^6^ The iFR was also shown to be faster to perform and have less adverse events compared with FFR.^10-12^

However, whether these results from randomized studies, suggesting that iFR can be used as surrogate for FFR in percutaneous interventions in CAD, can be transposed to clinical practice is still uncertain. Besides, factors like the costs of equipment, inadequate reimbursement, the interventional cardiologist preference, signs and symptoms reported by patients, and the costs and risks associated with adenosine treatment may limit the use of both methods. The use of iFR in a routine manner in patients with multi-vessel diseases and in non-culprit lesions in acute myocardial infarction (AMI) patients still need to be investigated.^13^

The present study aimed to evaluate the additional contribution of iFR to the therapeutic decision-making. The iFR was used in coronary disease patients in which the correlation between obstructive atherosclerotic disease and myocardial ischemia had not been clearly established by other conventional diagnostic methods.

## Methods

The study was approved by the ethics committee of Marcilio Dias Naval Hospital (approval number CAAE: 58741716.0.000.5256).

We studied patients older than 18 years, of both sexes, consecutively referred for percutaneous treatment between May 2014 and March 2018. All patients were referred for invasive investigation of myocardial ischemia and decision-making process by the Heart Team, composed by interventional cardiologists, clinical cardiologists and cardiovascular surgeons.

All patients with moderate (41-70% stenosis) or severe (71%-90%) stenosis according to coronary angiography were included. In all these patients there were doubts about the degree of obstruction, determined by coronary angiography, and its correlation with the presence of ischemia determined by non-invasive methods including ergometric test, myocardial scintigraphy and stress echocardiography.

The study population was composed of a wide variety of patients - patients with suspected or confirmed diagnosis of stable CAD but inconclusive diagnosis of myocardial ischemia using non-invasive methods; non-ST-elevation myocardial infarction patients in which the culprit artery had been treated, and invasive functional analysis had been performed in another coronary vessel with moderate-to-severe lesion by angiography; ST-elevation myocardial infarction patients in which invasive functional analysis of moderate-to-severe non-culprit lesion had been performed at least 5 days after the acute event.

The iFR was performed using the Volcano S5 Imaging System (San Diego, California, USA). The 0,014” Primewire Prestige® Pressure Guide Wire was used in 2014, and the 0,014” Verrata Pressure Guide Wire, substitute for the previous version, used in 2015. A guiding catheter was used to advance the guide wire through the lesion.^14-16^

All procedures were performed according to good practice guidelines for iFR measurements, as follows - the 0.014’’ guidewire was stabilized before handling by infusion of 0.9% saline until completion of the circuitry where the catheter was packed, and connection of the catheter to the console; during this process, the device was kept in stable position until it was recognized by the console software. After the guide wire was introduced into the catheter, it was externalized through the proximal coronary segment, and the guide pressure equalized using a transducer. The transducer guide was then positioned about 3 cm below the lesion.^15^ Also, guide pressure equalization was confirmed at the end of each measure to ensure its stability.^16^ To confirm the stability of the results, three consecutive measures were performed for each lesion; in case of diverging values, the lowest value was considered for analysis. Intracoronary nitroglycerin (200 µg, bolus) was administered before the measures were performed.^16^

The iFR was considered positive for myocardial ischemia 0.89 or less.^12^

### Statistical analysis

Categorical variables were described as numbers and percentages. Age (continuous variable) was described as mean and standard deviation, and as minimum, median and maximum values. Normality of the variable age was confirmed by the Shapiro-Wilk test (p = 0.3663). Distribution of the variable iFR was not tested for normality, and described as median and interquartile range.

A logistic regression was initially performed using the elastic net,^17^ which is a variable selection method that identifies strongly correlated predictors. This method is particularly useful when the number of predictors (P) is much bigger than the number of observations (n). In this model, the requirement of a stent was the outcome variable, and the independent variables were age, sex, comorbidities (such as systemic arterial hypertension, diabetes mellitus, dyslipidemias, smoking, family history, obesity and previous AMI). Two logistic regression models were built using the variables selected by the elastic net. In addition, we used a non-parametric classification tree,^18^ which is useful to detect possible interactions between predictors and provide easily interpreted visual information. The end nodes show the bar graph for the variable ‘stenting’. Additionally, the ROC curve was used to evaluate sensitivity and 1-speciticity of the iFR cut-off, established by the classification tree. Box plots^19^ were constructed to depict the distribution of the iFR values for moderate and severe stenoses, considering the use of stents. Statistical calculations were performed using the R package.^20^ The partykit package of the R software was used for construction of the classification tree.^21,22^ A p-value < 0.05 was considered statistically significant.

## Results

### Characteristics of the patients

**Figure 1:**
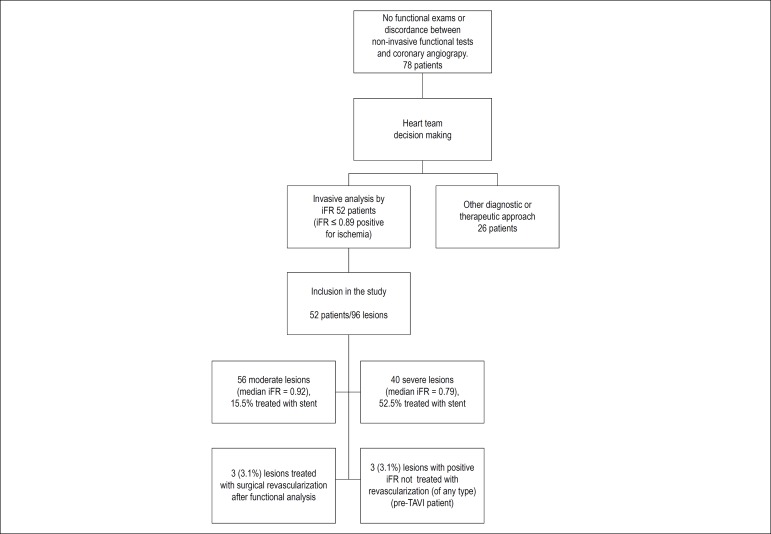
Flow chart of the study showing the heart team decision making for the stenotic lesions evaluated. iFR: instantaneous wave-free ratio; TAVI: transcatheter aortic valve implantation.

The iFR was used for assessment of 96 stenotic lesions of 52 patients, with a mean of 1.85 lesions/patient. Median iFR was 0.93 (0.855-0.97); 56 of them were classified as moderate stenosis (58.3%) and 40 of them as severe (41.7%) stenosis. Figure 1 shows the study flowchart. Thirty percent of the lesions were treated with stent placement, and in 6.2% of them, despite the presence of ischemia confirmed by functional analysis, the first therapeutic choice was other than stent placement - revascularization surgery due to the coronary anatomy (3.1%) and transcatheter aortic valve implantation (TAVI) (3.1%) - these therapeutic decisions were made by the Heart Team.

Regarding the localization of the stenotic lesions, 52 lesions were located in the anterior descending artery (54.1%), 11 in the circumflex artery (11.4%), 9 in the right coronary artery (9.3%), 10 in the diagonal branch (10.4%), 9 in the marginal branch (9.3%), 1 in the left posterior descending coronary artery (1.1%), 1 in the right posterior descendent artery (1.1%), 1 in the intermediate artery (1.1%), 1 in the posterior ventricular branch (1.1%), and 1 in the left main (1.1%). Characteristics of the patients are described in Table 1. There was a predominance of men and a high frequency of coronary risk factors, especially diabetes mellitus and smoking. The frequency of clinical manifestations was not different between chronic CAD and acute CAD patients. Most patients showed significant lesion in only one vessel, and approximately two thirds of them were not treated with stent placement.

**Table 1:** Characteristics of the patients

Variables	n (%)
Number of patients	52 (100%)
**Age**	
Mean ± SD	66.85 ± 11.27
Median (minimum, maximum)	66.5 (41, 86)
**Sex**	
Female	14 (26.9%)
Male	38 (73.1%)
Arterial hypertension	45 (86.5%)
Diabetes mellitus	22 (42.3%)
Dyslipidemia	36 (69.2%)
Smoking	17 (32.7%)
Family history of coronary artery disease	11 (21.2%)
Obesity	3 (5.8%)
Previous infarction	7 (13.5%)
**Clinical manifestation**	
Stable angina	19 (36.5%)
Myocardial acute infarction	21 (40.4%)
Others	12 (23.1%)
**Moderate stenoses**	
Without stenosis	16 (30.8%)
**With stenosis**	
1 lesion	18 (34.6%)
2 lesions	16 (30.8%)
3 lesions	2 (3.8%)
**Severe stenoses**	
Without stenosi	25 (48.1%)
**With stenosis**	
1 lesion	16 (30.8%)
2 lesions	9 (17.3%)
3 lesions	2 (3.8%)
**Stents**	
Without stent	30 (57.7%)
**With stent**	
1 stent	15 (28.8%)
2 stents	6 (11.5%)
3 stents	1 (1.9%)

SD: standard deviation.

### Statistical modelling and graphic analysis

Two logistic regression models were constructed to evaluate the need for stent placement. Model 1 was implemented using the variables selected by the elastic net - diabetes mellitus, dyslipidemia, presence of moderate stenosis and positive iFR. Model 2 was composed by the variables that showed statistical significance in the previous model - presence of moderate stenosis and positive iFR. Both dyslipidemia and diabetes mellitus lost statistical significance in the second model (Table 2).

**Table 2:** Logistic regression models

	Variable	Estimative	Standard error	Odds ration (95%CI)	p
Model 1	(Intercept)	7.8161	3.0611		0.0107
	Diabetes mellitus	0.4511	0.6360	1.570 (0.451; 5.461)	0.4782
	Dyslipidemia	0.9722	0.7391	2.644 (0.621; 11.256)	0.1884
	Moderate stenosis	-1.5000	0.5819	0.223 (0.071; 0.698)	0.0099
	iFR	-9.7182	3.4198	0.000 (0.000; 0.049)	0.0045
Model 2	(Intercept)	9.7209	2.8715		0.0007
	Moderate stenosis	-1.2414	0.5389	0.289 (0.100; 0.831)	0.0212
	iFR	-10.9861	3.2441	0.000 (0.000; 0.010)	0.0007

CI: confidence interval.

**Figure 2:**
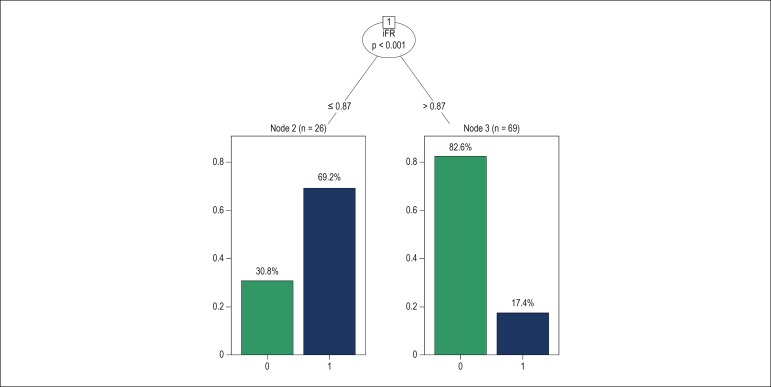
Classification tree for the logistic regression model 1; stent placement was observed in 69.2% of patients with iFR (instantaneous wave-free ratio) ≤ 0.87; and in 17.4% of patients with iFR > 0.87.

**Figure 3:**
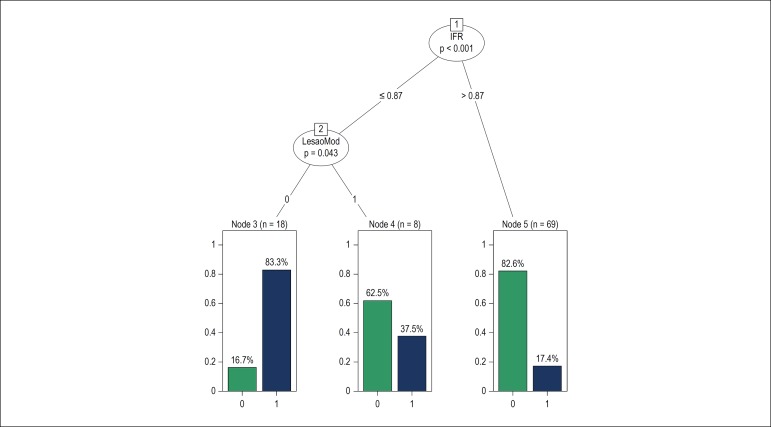
Classification tree for the logistic regression model 2; stent implantation was observed in 7.5% of patients with moderate stenosis and iFR (instantaneous wave-free ratio) ≤ 0.87.

Classification trees were developed to evaluate interactions between the predictors identified by logistic regression and facilitate their interpretation. (Figures 2 and 3). An iFR ≤ 0.87 was statistically associated with the occurrence of stent implementation, in nearly 37.5% of moderate stenotic lesions.

**Figure 4:**
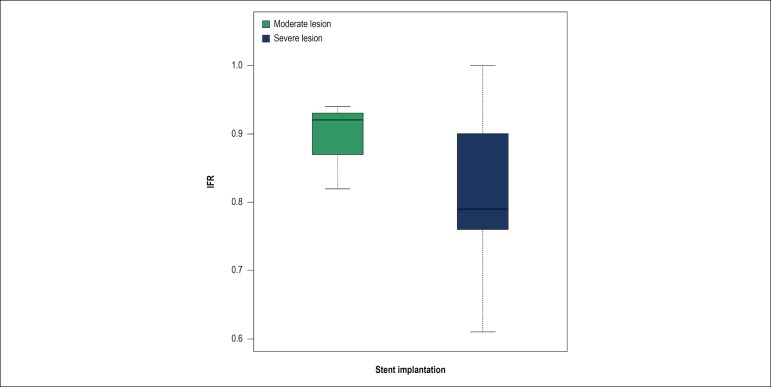
Box Plot of the iFR (instantaneous wave-free ratio) values for moderate and severe lesions considering the presence of stents. Median iFR was 0.92 (0.82-0.94) in moderate lesions and 0.79 (0.61-1.00) in severe lesions.

Figure 4 shows the box plot of the distribution of iFR values for moderate and severe lesions treated with stent placement. Among these, median iFR was 0.92 (0.82-0.94) for moderate lesions and 0.79 (0.61-1.00) for severe lesions, *i.e*., there was a higher variability in iFR values in severe lesions.

**Figure 5:**
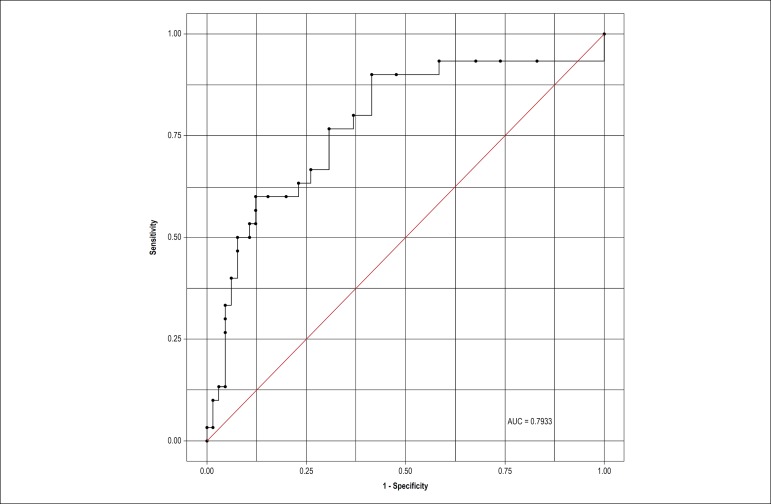
ROC curve for the iFR (instantaneous wave-free ratio); a sensitivity of 0.57 and 1-specificity of 0.88 was observed for the iFR cut-off point of 0.87, obtained from the classification tree. AUC: area under the curve.

The ROC curve evaluated sensitivity and 1-specificity of the iFR cut-off determined using the classification tree. Figure 5 depicts the ROC curve for the iFR, with an area under the curve of 0.7933 (95%CI, 0.6918-0.8949). A sensitivity of 0.57 and a 1-specificitity of 0.88 were obtained for an iFR cut-off of 0.87.

## Discussion

Previous studies have validated the iFR method in comparison with the FFR. the iFR was shown to be non-inferior to the FFR for composite outcomes in the DEFINE FLAIR study and for all-cause mortality, non-fatal AMI, and unplanned revascularization in the iFR-SWEDEHEART study after one-year follow-up. It is worth pointing out that in the iFR-SWEDEHEART trial, 17.5% of the patients treated had acute coronary syndrome.^7,23^ There are no randomized studies comparing iFR-guided revascularization *versus* medical therapy. Also, there is no strong evidence for the use of this new technique in AMI-related lesions or extrapolation of the outcomes to follow-up periods longer than one year. However, in a recent European guideline, a Class I recommendation with a level of evidence A has been issued to the iFR for intermediate lesions with no documentation of previous ischemia.^3^

The analysis of coronary physiology as a prerequisite for the prognostic assessment of moderate stenosis will be probably be incorporated to clinical practice, especially considering the iFR as an alternative to the FFR. As compared with the FFR, iFR is easier and faster to be performed, and prevent the side effects caused by intravenous infusion of vasodilators, especially CAD with acute clinical manifestations.^13^

In this context, this study corroborates previous findings of the literature,^9,24^ showing that, in situations where there were disagreements between anatomic and functional methods, moderate stenotic lesions in coronary angiography were reclassified, preventing stent implantation in 58% of the cases.

It is of note that the use of iFR helped in the therapeutic decision-making process, for stent placement, in moderate stenotic lesions in patients with stable CAD, and in non-culprit lesions of STEMI and non-STEMI patients. The combined analysis of the DEFINE-FLAIR and the iFR- SWEDEHEART studies,^13^ involving 440 patients with acute coronary syndrome, demonstrated a relative advantage of the iFR over FFR in these patients, but more robust studies are needed to confirm this. In the iFR-SWEEDHEART study, 38% of the patients had acute coronary syndrome, 17% of them with AMI without ST elevation, and 21% with unstable angina. The DEFINE-FLAIR trial, however, also included patients with AMI with ST elevation, 3.9% in the iFR group and 3.4% in the FFR group, in which the non-culprit vessel was analyzed at least 48 hours after the acute event.

Quantification of myocardial ischemia in the presence of serial lesions is challenging,^25^ as it is frequently seen in the descending coronary artery (DA), where the FFR has not been validated. In our study, 8 patients (15%) showed two or three serial lesions in the DA, with a total of 17 lesions analyzed by iFR. The ischemic component of the lesions was assessed, which was successfully treated with the placement of 5 stents, with no need to approach all the lesions. These data are corroborated by the iFR-GRADIENT Registry with 128 patients, in which the use of the iFR showed high accuracy in reclassifying the lesions in 31% of the cases.^26^

In the present study, the iFR cut-off of 0.87 showed high accuracy, with 0.57 sensitivity and 1-specificity of 0.88. The inclusion of severe lesions in our analysis may explain the lower sensitivity, as compared with literature data.

Discordance between FFR and iFR has been reported to occur in 20% of the cases and may be explained by differences in the hyperemic coronary flow velocity,^27^ which, in the presence of FFR (+) and iFR (-), is similar to that reported in non-stenotic vessels (by angiography). It is possible that such divergence is associated with pathophysiological mechanisms of the measures. Significant pressure differences caused by stenosis between resting and hyperemia indicate a considerable increase in flow, similarly to a coronary flow reserve (which is a directly measured parameter) greater than 2.0. In this context, the presence of an iFR > 0.90 and an FFR < 0.80 has been associated with a coronary flow reserve not limited by flow.^28^

In the present study, an iFR > 0.70 was found in the moderate lesions, and a higher variability was observed in severe lesions (0.61-1.00), mostly treated with stent placement. Such variability may be due flow changes associated with collateral supplied by microcirculation, more commonly seen in chronic lesions and in vessels that the irrigated area is not significant. In addition, there were 23 lesions in diagonal, marginal, posterior descending and posterior ventricular branches, corroborating previous hypothesis. Recently, the iFR/FRR-guided assessment has been suggested in complete revascularization in coronary three-vessel disease, venous grafts, and grafts in the circumflex system.^29^

The logistic regression models and the classification tress enabled the identification of the variables more frequently related with the coronary flow reserve. Diabetes mellitus, dyslipidemia, the presence of moderate stenosis and an iFR lower than 0.87 were predictors of stent implantation in moderate and severe lesions of CAD patients, in which results obtained from non-invasive tests and those of coronary angiography were discordant. However, when the model was constructed with significant variables only, only iFR < 0.87 and the presence of moderate stenosis remained in the model, indicating the importance of a functional analysis in this group of patients.

The main limitation of this study is the lack of both short-term and long-term follow-ups, which would allow us to evaluate whether there was an improvement in the clinical outcomes of the patients. Although a mere visual estimation of the lesion is a known limitation because of interobserver variation, it in fact reflects real-world clinical practice. The primary objective of the study was achieved - we showed the additional contribution of the iFR to the therapeutic decision making in moderate and severe coronary disease, when the correlation between obstructive coronary artery disease and myocardial ischemia is not clearly defined by conventional diagnostic methods.
